# Live birth rates in day 5 fresh versus vitrified single blastocyst transfer cycles: A cross-sectional analysis

**DOI:** 10.18502/ijrm.v21i3.13200

**Published:** 2023-04-14

**Authors:** Violet Kieu, Alex Polyakov, Genia Rozen, Daniel Lantsberg, Catharyn Stern, Wan Tinn Teh

**Affiliations:** ^1^Reproductive Services Unit, Royal Women's Hospital, Melbourne, VIC, Australia.; ^2^Department of Obstetrics and Gynaecology, The University of Melbourne, Melbourne, VIC, Australia.; ^3^Melbourne IVF, Melbourne, VIC, Australia.

**Keywords:** Blastocyst, Live birth, In vitro techniques, Pregnancy rate, Vitrification, Reproduction.

## Abstract

**Background:**

The use of frozen embryo transfers (FET) in assisted reproduction has increased worldwide. Controlled ovarian hyperstimulation in a fresh transfer may impair endometrial-embryo synchronicity. However, there is conflicting evidence on live birth rates (LBR) and clinical pregnancy rates (CPR).

**Objective:**

To compare LBRs and CPRs between single autologous day 5 fresh vs. vitrified blastocyst transfer cycles, to investigate the impact of controlled ovarian hyperstimulation on embryo-endometrium asynchrony.

**Materials and Methods:**

A large cross-sectional analysis of 6002 embryo transfers (ET) comprised 3774 fresh and 2228 FET cycles from 2016 to 2019. Multivariate and subgroup analysis were performed for high responders ( > 20 oocytes).

**Results:**

Univariate analysis showed no difference in LBR (28.3% vs. 27.4%, p = 0.43) and CPR (32.2% vs. 30.9%, p = 0.30); however, multivariate analysis demonstrated significantly lower LBR (OR 0.864, p = 0.046, 95% CI 0.749-0.997) and CPR (OR 0.852, p = 0.024, 95% CI 0.742-0.979) in FET compared to fresh ETs. Younger participant age, previous in vitro fertilization pregnancy, advanced blastocyst expansion, higher trophectoderm quality, and lower cumulative number of ETs all improved the odds of LBR and CPR. Conventional in vitro fertilization, rather than intracytoplasmic sperm injection, improved CPR but not LBR. Body mass index affected neither LBR nor CPR. In the subgroup, multivariate analysis of high responders showed no difference in LBR or CPR.

**Conclusion:**

This study demonstrates relatively higher LBR and CPR of nearly 14% for fresh ETs compared to FETs, in multivariate analysis. A universal freeze-all strategy, without appropriate indication, may lead to suboptimal outcomes. In high responders, freeze-all cycles may be beneficial, as outcomes appear similar.

## 1. Introduction

The use of frozen embryo transfers (FET) in assisted reproduction has increased worldwide (1-5). Higher numbers of freeze-all cycles, a move toward single embryo transfer (SET), improved survival rates of cryopreserved embryos with vitrification (6) and preimplantation genetic testing for aneuploidy (PGT-A) (1) have contributed to this trend (3).

In the Australia and New Zealand Assisted Reproductive Database, autologous freeze-all cycles have increased from 13.7-32.6% (5970/43,579 vs. 17,939/55,032) from 2014 to 2020, with a 45.5% increase in thawed FETs (24,395 to 35,497). Conversely, fresh embryo transfers have decreased from 65.0% to 43.1%, comprising less than half of all initiated stimulation cycles (28,333/43,579 vs. 23,739/55,032) (1, 2). Similar trends have also been observed in other industrialized regions such as Europe, Asia, and North America (3-5).

It is known that controlled ovarian hyperstimulation (COH) with pituitary suppression induces a more histologically advanced endometrium than in natural cycles (7, 8) with dyssynchronous glandular and stromal differentiation (9, 10). Proponents of freeze-all suggest that COH in a fresh transfer impairs endometrial-embryo synchronicity, which would be avoided in a subsequent freeze-thaw cycle (11). Furthermore, freezing may allow for risk reduction of ovarian hyperstimulation syndrome (OHSS) (12, 13) with an agonist trigger, fertility preservation, for trophectoderm biopsy in PGT-A, or participant choice (1).

Given the global rise in FET, we have undertaken this study to evaluate the freeze-thaw effect on pregnancy outcomes, compared with fresh transfers for day 5 blastocysts. This is important given the conflicting evidence on the live birth rate (LBR) and clinical pregnancy rate (CPR) outcomes of fresh vs. frozen Embryo transfer (ET) (14-20). A recent 2021 Cochrane review showed similar results in cumulative LBR between freeze-all and conventional in vitro fertilization/intracytoplasmic sperm injection (Odd ratio [OR] 1.08, 95% CI 0.95-1.22), with no clear evidence of difference (16). This included older papers reporting on cleavage stage embryos and slow freeze, whereas more recent publications report blastocysts and vitrification (14, 15). Blastocysts have been shown to have a higher LBR per embryo transfer compared to cleavage stage (21) and vitrification has demonstrated a significant improvement in embryo cryosurvival compared to slow freeze (relative risk [RR] = 1.59, 95% CI: 1.30-1.93; p < 0.001) (5). None of the studies in the Cochrane review were from our Australian and New Zealand region.

Our study aimed to use real world data of our large in vitro fertilization (IVF) participant database to compare LBRs and CPRs between single autologous day 5 fresh vs. vitrified blastocyst transfer cycles, to investigate the impact of COH on embryo-endometrium asynchrony, particularly in an Australian context given the paucity of published data.

## 2. Materials and Methods

### Study design and setting

This was a large retrospective cross-sectional analysis. A standardized data set from 2016 to 2019 was retrieved from our multi-laboratory Melbourne IVF participant database, in Melbourne, Australia. This included 6002 day 5 autologous SETs, either fresh or vitrified.

### Participants

Study inclusion criteria specified a maximum of 2 stimulated cycles per individual in their first or second cycle, where a cycle included ovarian stimulation and transfer of all embryos created from the cycle, fresh or frozen. Inclusion was limited to cycles using embryos transferred fresh on day 5 or vitrified on day 5. Exclusion criteria were maternal age > 46 yr, cleavage-stage embryos, slow-frozen blastocysts, > 1 blastocyst transferred, donor oocytes or embryos, PGT-A, and embryos cultured on beyond day 5 for FET.

Blastocysts were derived from participants undergoing conventional IVF or intracytoplasmic sperm injection (ICSI) cycles. For conventional IVF cycles, oocytes were inseminated with 10 million/ml sperm overnight and denuded of cumulus cells 15-16 hr post-insemination (HPI). For ICSI cycles, oocytes were denuded of surrounding cumulus cells using a hyaluronidase solution (SynVitro Hyadase, CooperSurgical, US).

Inseminated oocytes were cultured in G-TL media (Vitrolife AB, Sweden) in 12-well dishes (Vitrolife AB, Sweden) or EmbryoScope+/EmbryoScope dishes (Vitrolife AB, Sweden) and covered with Ovoil (Vitrolife). Embryos were cultured in a MINC (Cook Medical), EmbryoScope, or Embryoscope+. Incubator conditions were 5% O_2_, 6% CO_2_, and 89% N^2^ at 37 C. Fertilization check occurred at 16-18 HPI, and on day 5 of development (approx. 112-115 HPI), embryos were assessed for transfer or vitrification or cultured until day 6 or 7, where they were either vitrified or discarded accordingly. Vitrolife Rapidi was used as the vitrification device. Morphological assessment on day 5 included blastocyst expansion, inner cell mass (ICM), and trophectoderm quality (22, 23).

Our unit changed to the Gardner classification system during the study period. Thus, some embryos were classified with the old system of either simply `yes' or `no' for the presence of ICM. Blastocyst expansion grade was classified as follows: 1 (cavitating embryo), 2 (early blastocyst), 3 (full blastocyst), 4 (expanded blastocyst), 5 (hatching blastocyst), and 6 (hatched blastocyst). The ICM and trophectoderm quality were classified as either A (good), B (fair), or C (poor). Embryos graded Gardner 1 or above were considered suitable for fresh ET, whereas the criteria for freezing embryos was grade 2 or above, later changed to grade 3BB or above.

For the vitrification-warm protocol, blastocysts were warmed on the morning of transfer and assessed for percentage of cell survival and degree of expansion. Blastocysts were only transferred under ultrasound guidance if assessed to have > 50% cell survival. Warmed embryos were replaced in a natural, modified natural, ovulation induction, or artificial hormone replacement cycle.

### Variables 

Clinical outcomes were LBR and CPR, with LBR defined as `the complete expulsion or extraction from its mother of a baby, irrespective of the duration of the pregnancy, which, after such separation, breathes or shows any other evidence of life' (1, 24), and CPR was defined as ultrasound evidence of an intrauterine sac, with or without a fetal heart (1).

Subgroup analysis of high responders, defined as > 20 oocytes collected, was selected due to possible increased embryo-endometrium asynchronicity with an altered hormonal milieu, as well as being a clinical indication to convert to a freeze-all cycle to avoid the risk of OHSS (25). Furthermore, in previous studies fresh vs. frozen outcomes for high responders have been conflicting (12, 26).

### Ethical considerations

This study was approved by the Melbourne IVF Human Research and Ethics Committee, Melbourne, Australia (HREC ID: 71/19-MIVF).

### Statistical analysis 

The Software for Statistics and Data Science, version 9.2, StataCorp, College Station, Texas, USA (STATA), was used for statistical analysis. Univariate analyses of participant characteristics and pregnancy outcomes were performed using the Chi-squared test for proportions and the student's *t* test for continuous variables. Multivariate logistic regression was used to further evaluate the relationship between fresh vs. frozen ETs and clinical outcomes, controlling for other clinically relevant variables, including female age at the time of ovum pick up (OPU), previous IVF pregnancy, blastocyst expansion grade, trophectoderm quality, number of cumulative ETs, fertilization method and BMI. The p-value of ≤ 0.05 was statistically significant in the logistic regression analysis.

## 3. Results 

### Participants

The study included a total of 6002 embryo transfers, comprising 3774 fresh and 2228 FET cycles. The baseline characteristics were compared between the 2 cohorts of fresh vs. vitrified day 5 embryo transfers (Table I). The average number of eggs collected per participant overall were 12.66 oocytes.

### Descriptive data

The baseline characteristics between fresh and FET cycles were not significantly different for BMI, follicle-stimulating hormone (FSH) duration, or fertilization method. Women in the vitrified group were younger, had a lower starting dose of FSH in the cycle in which the embryo was created, had more cumulative ETs, and were more likely to have a previous IVF pregnancy (p < 0.001). Embryo quality was significantly higher in fresh transfers than frozen, defined by blastocyst expansion grade (p < 0.001) and trophectoderm quality (p < 0.001).

### Outcome data

Univariate analysis of LBR and CPR with fresh ET vs. FET (Table II) neither showed any differences in CPR (p = 0.30) and LBR (p = 0.43) nor in multiple pregnancy rate (p = 0.19). There were 10 stillbirths in the fresh ET group, one in the FET group, with 3 neonatal deaths in the fresh ET group compared to one in the FETs.

Due to the aforementioned change in embryo grading classifications during the study period, combining the 2 systems was not possible; hence ICM data has not been included in this analysis. After controlling for other clinically significant variables in a multivariate analysis (Table III), there was a 13.6% higher LBR in the fresh vs. frozen cohort (OR 0.864, p = 0.046, 95% CI 0.749-0.997) and 15% higher CPR (OR 0.852, p = 0.024, 95% CI 0.742-0.979). As expected, age was inversely associated with the odds of both CPR and LBR, with every 1 yr increase in age over 35 yr old associated with a 10% reduction in LBR ( < 0.001) and a 13% reduction in CPR (p < 0.001). Previous IVF pregnancy was strongly associated with both outcomes, increasing the odds ratio for both LBR and CPR by more than 50% (p < 0.001).

Higher blastocyst expansion grade increased the odds of CPR by 32% (p < 0.001) and LBR by 27% (p < 0.001), with improved trophectoderm quality, similarly increasing the odds of CPR by 26% (p < 0.001) and LBR by 27% (p < 0.001). Previous cumulative ET number was inversely associated with CPR and LBR (p < 0.001). The fertilization method of IVF rather than ICSI statistically improved the odds of CPR by 14% (p = 0.013) but not LBR (p = 0.138). BMI was neither a significant predictor of CPR or LBR, with p = 0.36 and 0.49 respectively.

### Subgroup analysis for high responders

Subgroup analysis for high responders (n = 795) showed no difference in multivariate analysis for fresh vs. frozen transfers (Table IV), age, trophectoderm quality, cumulative ET, fertilization method, or BMI. The average number of oocytes collected per cycle was 26.89.

In high responders, a previous IVF pregnancy was again a strong predictor of pregnancy outcomes, increasing odds of CPR by more than 60% (p = 0.041) and LBR by 80% (p = 0.015). Higher blastocyst expansion grade improved odds by greater than 40% for both CPR (p < 0.001) and LBR (p < 0.001), with less cumulative ETs improving OR of both CPR (p = 0.044) and LBR (p = 0.014) by greater than 15%.

**Table 1:** Baseline characteristics


**Variables**		**Fresh ET** **(n = 3774)**	**FET** **(n = 2228)**	**P-value**
Age* (yr)		35.8 ± 4.2	34.5 ± 4.0	< 0.001
BMI* (kg/m^2^)		24.9 ± 6.9	24.6 ± 6.1	0.08
FSH start dose* (IU)		228.7 ± 81.6	205.3 ± 75.1	< 0.001
FSH duration* (days)		10.03 ± 6.4	10.4 ± 5.6	0.47
OPU number**				
	**First**	2705 (71.7)	1783 (80)	
	**Second**	1069 (28.3)	445 (19.97)	
Cumulative ET**				
	**1**	2919 (77.3)	380 (17.1)	
	**2**	412 (10.9)	947 (42.5)	
	**3**	247 (6.5)	478 (21.5)	
	** ≥ 4**	196 (5.2)	423 (19)	< 0.001
	Previous IVF pregnancy**	294 (7.8)	462 (20.7)	< 0.001
Fertilization methods**				
	**ICSI**	2,365 (62.7)	1413 (63.4)	
	**IVF**	1409 (37.3)	815 (36.6)	0.56
Blastocyst expansion grade**				
	**5 & 6 (Hatched & Hatching)**	340 (9)	119 (5.3)	
	**4 (Expanded)**	1671 (44.3)	1143 (51.3)	
	**3 (Full)**	862 (22.8)	703 (31.6)	
	**2 (Early)**	478 (12.7)	222 (10)	
	**1 (Cavitating)**	423 (11.2)	41 (1.8)	< 0.001
Trophectoderm quality**				
	**A**	1965 (66.4)	1201 (57.9)	
	**B**	909 (30.7)	804 (38.8)	
	**C**	85 (2.9)	70 (3.4)	< 0.001
	**A**	541 (33.1)	219 (17.6)	
	**B**	204 (12.5)	121 (9.7)	
	**C**	10 (0.6)	4 (0.3)	
	**No**	1 (0.1)	2 (0.2)	
	**Yes**	878 (53.7)	899 (72.2)	< 0.001
*Data presented as Mean ± SD. **Data presented as n (%). Chi-squared test used to compare proportions, Student's *t* test used for continuous variables. BMI = Body mass index, FSH = Follicle-stimulating hormone, IU = International units, OPU = Oocyte pick-up, N = Number, % = Percent, ET = Embryo transfer, FET = Frozen embryo transfer, ICSI = Intra-cytoplasmic sperm injection, IVF = In vitro fertilization, ICM: Inner cell mass				

**Table 2:** Univariate analysis with CPR and LBR outcomes


**Variables**	**Fresh ET (n = 3774)**	**FET (n = 2228)**	**P-value**
**Clinical pregnancy **	1214 (32.2)	688 (30.9)	0.30
**Live birth**	1069 (28.3)	610 (27.4)	0.43
**Multiple pregnancies **	26 (2.4)	9 (1.5)	0.19
CPR = Clinical pregnancy rate, LBR = Live birth rate, ET = Embryo transfer, FET = Frozen embryo transfer. Data presented as n (%). Chi-squared test used for comparisons			

**Table 3:** Multivariate analyses


**Variables**		**Odds ratio**	**[95% CI]**		**P-value**
**Live birth rate**					
	**Cycle type (fresh ET vs. FET)**	0.864	0.749	0.997	0.05
	**Age (yr)**	0.871	0.845	0.899	< 0.001
	**Previous IVF pregnancy**	1.530	1.247	1.878	< 0.001
	**Blastocyst expansion grade**	0.725	0.658	0.799	< 0.001
	**Trophectoderm quality**	0.721	0.637	0.816	< 0.001
	**Cumulative ET**	0.862	0.796	0.933	< 0.001
	**Fertilization method (IVF vs. ICSI)**	0.909	0.801	1.031	0.14
	**BMI (kg/m^2^)**	1.003	0.994	1.013	0.49
**Clinical pregnancy rate**					
	**Cycle type (fresh ET vs. FET)**	0.852	0.742	0.979	0.02
	**Age (yr)**	0.901	0.876	0.928	< 0.001
	**Previous IVF pregnancy**	1.526	1.251	1.861	< 0.001
	**Blastocyst expansion grade**	0.673	0.613	0.740	< 0.001
	**Trophectoderm quality**	0.735	0.653	0.829	< 0.001
	**Cumulative ET**	0.882	0.817	0.952	< 0.001
	**Fertilization method (IVF vs. ICSI)**	0.856	0.757	0.967	0.01
	**BMI (kg/m^2^)**	1.004	0.995	1.014	0.36
ET = Embryo transfer, FET = Frozen embryo transfer, IVF = In vitro fertilization, ICSI = Intracytoplasmic sperm injection, BMI = Body mass index. 95% CI = 95% confidence interval. Logistic regression analysis was used, with live birth and clinical pregnancy being the outcome variables					

**Table 4:** Multivariate analysis in high responders


**Variables**		**Odds ratio**	**[95% Cl]**		**P-value**
**Live birth rate**					
	**Cycle type (fresh ET vs. FET)**	1.153	0.804	1.653	0.439
	**Age (yr)**	0.992	0.952	1.032	0.686
	**Previous IVF pregnancy**	1.814	1.121	2.935	0.015
	**Blastocyst expansion grade**	0.575	0.454	0.728	0.000
	**Trophectoderm quality**	0.979	0.689	1.392	0.907
	**Cumulative ET**	0.808	0.681	0.959	0.014
	**Fertilization method (IVF vs. ICSI)**	0.921	0.663	1.280	0.622
	**BMI**	1.001	0.974	1.029	0.929
**Clinical pregnancy rate**					
	**Cycle type (fresh ET vs. FET)**	1.147	0.803	1.640	0.450
	**Age (yr)**	1.005	0.966	1.047	0.802
	**Previous IVF pregnancy**	1.630	1.021	2.603	0.041
	**Blastocyst expansion grade**	0.550	0.437	0.693	0.000
	**Trophectoderm quality**	1.189	0.855	1.655	0.303
	**Cumulative ET**	0.846	0.719	0.995	0.044
	**Fertilization method (IVF vs. ICSI)**	0.991	0.715	1.375	0.961
	**BMI**	1.007	0.980	1.034	0.632
IVF = In vitro fertilization, BMI = Body mass index, ET = Embryo transfer, FET= Frozen embryo transfer, ICSI = Intracytoplasmic sperm injection. 95% CI = 95% confidence interval. Logistic regression analysis was used, with live birth and clinical pregnancy being the outcome variables					

## 4. Discussion

In this large cross-sectional analysis of 6002 autologous SETs, we demonstrated that fresh day 5 blastocyst transfers were associated with an increased LBR and CPR compared to FET in multivariate analysis. Having a FET significantly decreased the odds of LBR by nearly 14% when accounting for age, previous IVF pregnancy, blastocyst expansion grade, trophectoderm quality, previous cumulative ETs, fertilization method, and BMI (OR 0.864, p = 0.046) (Table III).

Our results support our current clinical practice of transferring a fresh embryo, where possible, and utilizing the freeze-all strategy judiciously. Contemporary clinical practice had moved toward blastocyst transfers and vitrification. Australia and New Zealand Assisted Reproductive Database reports blastocyst transfer cycles had increased from 67.5% in 2014 to 89.4% in 2020 and vitrification from 85.6–95.7% (1).

Our findings align with previous cohort studies and a randomized controlled trial (RCT) (18-20). A large population cohort study of 337,148 IVF cycles in the United Kingdom (UK) demonstrated that freeze-all and FET strategy were associated with a lower cumulative LBR when adjusted for age, cycle number, cause of infertility, and ovarian response (RR 0.80, 95% CI 0.78-0.83), where elective-freeze all was not recommended (18).

A recent pragmatic 2-arm RCT was conducted across 18 clinics in the UK (19). A total of 619 couples were randomized with the first ET from a freeze-all or fresh cycle, with no difference in LBR (28.3% vs. 34.3%; RR 0.83, 99% CI 0.65 to 1.06); however, there was a tendency to favor fresh transfer (19). Furthermore, freeze-all was unlikely to be cost effective, given more monitoring visits and ultrasound costs, even when accounting for higher OHSS rates (3.6% FET vs. 8.1% fresh) (19).

In contrast to our results, Stormlund et al. showed no statistical difference in LBR for freeze-all vs. fresh ETs (27.4% vs. 28.7%, RR0.98, 95% CI 0.87-1.10, p = 0.83) in RCT of 460 women. This RCT was limited to women aged 18-39 yr with regular menstrual cycles and good ovarian reserve (14). Women with irregular menses, PCOS, age > 40 yr, and low-ovarian reserve were excluded. A relatively large number of protocol deviations were also reported in this RCT (13.5% had protocol deviation in the fresh arm), mostly due to the risk of OHSS and subsequent freeze-all in the fresh arm. A more restrictive subject group and stimulation protocol in the RCT may explain our results differences (18). Furthermore, a cohort study from Iran of 1419 cycles demonstrated no difference between FET and fresh transfer LBR (65.6% vs. 70.4% respectively) (20).

On the other hand, another RCT by Wei et al. (15) involving 825 women in China reported higher LBR for freeze-all than fresh ET (CPR 50% vs. 40%, RR1.26, 95% CI 1.14-1.41, p < 0.0001). This study had a cohort of good prognosis participants with an overall mean age of 28.8 yr (21-35 yr), 14 oocytes were retrieved per woman, and an average BMI of 22. Unlike their population, our women had a higher mean age (fresh ET 35.8 yr and FET 34.5 yr), 12.7 was the average number of oocytes retrieved overall, and BMI of 24.9 and 24.6, respectively. Thus our results are dissimilar to Wei, who had a different participant demographic and required inclusion criteria of > 4 embryos on day 3, suggesting better prognosis participants, reflected in their relatively high LBR in both arms. As such, their results are not generalizable to our population. Notably, their FET cohort was associated with a higher risk of preeclampsia PET (RR 3.13, p = 0.29) (15).

A secondary finding of our study was that there was no statistical difference in the CPR (p = 0.450) and LBR (p = 0.432) between fresh vs. frozen ET in subgroup multivariate analysis of high responders with > 20 oocytes at OPU, though a nonsignificant trend toward improved outcomes with frozen transfer was detected (Table IV). The loss of improvement in LBR and CPR in fresh transfers in high responders may suggest a negative impact of increased hormonal milieu and subsequent impaired endometrial receptivity (11, 12). Clinically, this may indicate that if high responders are converted to freeze-all cycles to avoid OHSS, it may not be detrimental to LBR or CPR to do so (12, 13).

An RCT of high responders also showed a similar trend toward improved outcomes in FET that was not statistically significant. In this study, only CPR was reported, with a mean number of 2 embryos transferred (1.98 fresh and 1.90 FET). A large number of thawed embryos were lost, with a mean of 13.8 embryos thawed compared to 11.5 mean survival, indicating a 16.7% loss of frozen-thawed embryos. There was higher embryo quality in fresh transfers and suggested less impaired endometrium in frozen transfers, with a trend that did not reach the statistical significance of higher CPR in FET vs. fresh cycles (79.6% vs. 65.4%, p = 0.1109) (12). Our study included only a single ET, reported LBR, and on average, had a higher number of oocytes collected 26.9, compared to their fresh 19.3 and FET 20.9 oocytes retrieved.

A recent meta-analysis of 8 RCTs has also suggested that freeze-all and FET were associated with a higher probability of LBR in high responders (RR 1.18, 95% CI 1.06-1.31), but not in normal responders (RR 1.13, 95% CI 0.90-1.41) (13). Differences in results between studies of fresh and FET could be explained by differences in participant populations (e.g., age, high responders and preexisting medical conditions such as PCOS) and type of stimulation and subsequent endometrial maturation effects.

The strength of our paper is that we had a large sample size for analysis due to our comprehensive database, specific to the Australian population. We have limited our analysis to the first 2 stimulation cycles, so those with multiple failed cycles were not overrepresented in the dataset. Furthermore, multivariate analysis was performed to account for potential confounders.

The retrospective nature of this analysis is the main limitation of our study, alongside the order in which the embryos were transferred; for example, the `best-graded' embryo was transferred fresh. However, all embryos were graded, so we could quantify this and perform multivariate analysis accordingly. Furthermore, we acknowledge that our study was not comparing elective freeze-all vs. fresh. Before the Gardner classification scale was introduced, ICM was only documented as being present as 'Yes' or 'No'. So, `Yes' could either be Gardner grade A or B, and it was impossible to reclassify them retrospectively. As a result, the study's limitation is that ICM could not be included in the multivariate analysis. Another limitation is that the endometrial preparation for the thaw cycle varied with clinician practices, thus including both natural and artificial cycle preparation (27).

## 5. Conclusion

In conclusion, this large retrospective study of day 5 fresh vs. vitrified frozen blastocyst transfer does not demonstrate the superiority of frozen over fresh ET. As such, we would caution against an indiscriminate application of the freeze-all strategy to all participants. Considering the potential loss of embryos in the freeze-thaw process, it is likely that cumulative pregnancy outcomes may be compromised with more widespread implementation of the elective freeze-all strategy; however, RCTs are required to confirm this. Moreover, in our subgroup analysis of high responders, no statistically significant difference was observed between fresh and FET outcomes of LBR and CPR, suggesting that in women with > 20 oocytes at OPU freeze-all does not compromise pregnancy outcomes is an important OHSS risk mitigating strategy.

##  Conflict of Interest

The authors declare that they have no conflict of interest.
